# Age-Period-Cohort Analysis of Lung Cancer Mortality in Japan, 1960-1995

**DOI:** 10.2188/jea.11.151

**Published:** 2007-11-30

**Authors:** Hideto Takahashi, Masafumi Okada, Katsumi Kano

**Affiliations:** 1 Institute of Community Medicine, University of Tsukuba, Ibaraki, Japan.; 2 Graduate school of Medicine, University of Tsukuba, Ibaraki, Japan.

**Keywords:** age-period-cohort analysis, lung cancer

## Abstract

The mortality data on lung cancer in Japan from 1960 to 1995 was analysed based on an age-period-cohort (APC) model. Though the APC model has an ‘identifiable problem’ caused by the relationship of age, period and cohort parameters, non-linear components of them revealed their original (separated) effects. They were: (1) non-linear age effects had a peak in 55-59 and 60-64 years old in males and 50-54 in females, (2) non-linear period effects were very small in both genders, (3) non-linear age and period effects were small enough to neglect compared with their linear effects, and (4) there were five parts of trends in Japanese lung cancer mortality in both genders in the non-linear birth cohort effects. The 1961-65 birth cohort effect seemed to increase differently from previous birth years. This trend should be monitored carefully.

## INTRODUCTION

In Japan, deaths caused by cancer have been increasing, and cancer has been the leading cause of death since 1981^1^^)^. Lung cancer especially is the leading cause of death in males and has the third highest mortality rate in females among all forms of cancer at the present. To reveal the temporal trends of age, period and birth cohort effects is of importance for providing clues or hypotheses for its aetiology. Components of age, period and birth cohort of death are three separate temporal factors related to the mortality of the cancer.

Each component bears a different biological meaning in the process of carcinogens with the multi-stage model^2^^, ^^3^^)^. For example, exposure to early stage carcinogens (initiators) will often introduce a birth cohort effect, whereas late-stage carcinogens (promoters) or diagnostic and therapeutic improvements will produce a period effect. Standard cross-sectional analysis of the trend, however, cannot separate age and period effects. In general, conventional birth cohort analysis is a mainly graphical approach and only can provide combined effects of the three time factors mentioned above. To differentiate them, a log linear model has been developed in the past two decades and applied to the analysis of various forms of cancer.

The earliest use of this form of modelling was Kermack et al^4^^)^ and Frost^5^^)^, and early works of development of this model were briefly summarized in Osmond and Gardner^6^^)^. Though Kupper^7^^, ^^8^^)^ gave critiques of this model in the point of interpretation, this model has been developed by many authors. At present, the APC model is used for many studies^9^^-^^12^^)^ for its instructive usefulness.

In the APC model, the three time variables are not identifiable due to the exact linear relation:

birth year+age at death=calendar year at death (1)

Many methods have been developed to solve the problem, which are classified into roughly five groups at the present: (1) penalty function approach^6^^, ^^14^^)^, (2) individual records approach^15^^)^ (3) autoregressive models^16^^)^, (4) Bayesian approach^17^^-^^19^^)^ and (5) estimable function approach a^20^^, ^^13^^, ^^21^^, ^^22^^)^ (deviation from linearity, curvature, drift). Once non-overlapping cohort or individual records looked to overcome ‘nonidentifiability^23^^, ^^15^^)^, however, they did not solve it^24^^-^^26^^)^.

Estimable function approach was developed in three ways, (local) curvature approach^20^^)^ based drift model^27^^)^, non-linear component approach (deviation from linearity)^13^^)^ and extended curvature approach^22^^)^. In these three estimable function approaches, there is the relation where differences of successive non-linear components produce local curvatures. In the branch of this development, Osmond tried to predict future mortality based on the APC model^28^^)^ and Robertson and Bolye^29^^)^ tried a graphical approach. Tarone developed nonparametric evaluation of birth cohort trends^30^^)^ in the APC analysis. Recently, a few comparative studies recommended that the method based estimable function approach (5) was the most appropriate to the APC model^31^^, ^^32^^)^.

In this study, mortality data of lung cancer in Japan over the period of 1960 to 1995 was analysed. Separated age, period and birth cohort effects were analysed and are presented using the APC model based on non-linear ‘deviation from linearity’ by Holford^13^^, ^^33^^)^.

## MATERIALS AND METHODS

### Data Source

Lung cancer in this study was defined by the international detailed list code 162,163 (ICD-7) in 1960 and 1965, code 162 (ICD-8) in 1970 and 1975, code 162 (ICD-9) in 1980, 1985 and 1990, and code C33, C34 (ICD-10) in 1995. The cancer deaths data for the quinquennial years during 1960 to 1995 were obtained from Japanese Vital Statistics^1^^)^, summarized in a two-way, age by period contingency table with unequal person-years at risk in each cell in Table 1 in males and Table 2 in females. The death rates were calculated with the population data that were obtained from the eight successive national censuses of 1960, 1965, …, 1990 and 1995^1^^)^. Age was divided into 10 quinquennia; 30-34, 35-39, …, 75-79 years old. Person-years in each census year were estimated by the population data in that year.

**Table 1.** Lung cancer mortality rates (per 100,000) among Japanese males, 1960-1995.

Age(years)		Calender period										

		1960		1965		1970		1975	1980	1985	1990	1995
30-34	rate	0.694		0.772		0.769		0.870	0.872	0.947	1.002	1.165
	deaths (obs)	26		32		32		40	47	43	39	47
	expected	(26)		(29)		(27)		(37)	(54)	(49)	(38)	(47)
35-39	rate	1.122		1.788		1.609		1.862	2.495	2.895	3.289	2.597
	deaths	31		67		66		78	114	156	148	101
	expected	(47)	*	(73)		(78)		(76)	(105)	(149)	(131)	(102)
40-44	rate	2.726		3.627		4.551		4.894	5.027	6.120	6.750	6.448
	deaths	62		99		166		201	208	275	360	289
	expected	(88)	**	(113)		(170)		(191)	(190)	(255)	(352)	(301)
45-49	rate	7.666		8.451		8.469		11.432	11.203	11.128	12.925	14.311
	deaths	173		188		225		416	450	451	578	757
	expected	(180)		(199)		(251)		(393)	(448)	(435)	(567)	(765)
50-54	rate	15.779		18.731		20.141		20.831	26.365	25.242	22.977	25.946
	deaths	322		407		431		541	931	984	917	1140
	expected	(329)		(389)		(422)		(551)	(888)	(980)	(930)	(1183)
55-59	rate	30.463		32.013		40.666		43.886	48.837	53.609	53.365	47.119
	deaths	549		618		825		903	1218	1818	2018	1831
	expected	(549)		(674)	*	(772)		(882)	(1191)	(1854)	(1999)	(1858)
60-64	rate	46.537		66.335		71.533		79.665	96.021	98.821	112.972	104.537
	deaths	669		1078		1249		1533	1856	2321	3654	3761
	expected	(680)	*	(1040)		(1259)		(1545)	(1808)	(2385)	(3616)	(3788)
65-69	rate	71.373		93.119		124.959		139.352	157.167	179.061	190.242	202.257
	deaths	733		1135		1741		2179	2726	3171	4165	6042
	expected	(682)		(1111)		(1687)		(2182)	(2789)	(3207)	(4135)	(6100)
70-74	rate	86.942		118.505		156.627		210.485	252.723	284.503	300.337	316.056
	deaths	603		935		1501		2407	3316	4228	4675	6104
	expected	(587)		(914)		(1498)		(2426)	(3318)	(4212)	(4782)	(6031)
75-79	rate	81.761		126.142		160.900		238.261	329.139	403.130	419.712	454.564
	deaths	308		570		854		1635	2784	4018	5022	5702
	expected	(308)		(586)		(926)	*	(1651)	(2858)	(3939)	(5026)	(5600)

* P<0.05, **P<0.01. Both were calculated from (obs-exp)^2^/exp.

**Table 2.** Lung cancer mortality rates (per 100,000) among Japanese females, 1960-1995.

Age(years)	Calender period											

		1960		1965		1970	1975	1980	1985		1990	1995
30-34	rate	0.849		0.852		0.840	0.740	0.771	0.400		0.707	0.915
	deaths	32		35		35	34	41	18		27	36
	expected	(22)	*	(26)		(28)	(34)	(46)	(38)	**	(28)	(36)
35-39	rate	1.252		1.786		1.205	1.743	1.702	1.779		1.979	1.728
	deaths	41		67		49	73	78	95		88	66
	expected	(43)		(55)		(56)	(65)	(85)	(103)		(84)	(65)
40-44	rate	1.785		2.692		2.897	2.450	2.910	3.251		3.103	3.811
	deaths	49		87		106	100	121	149		164	169
	expected	(66)	*	(84)		(90)	(100)	(125)	(148)		(179)	(153)
45-49	rate	4.922		3.893		5.184	5.420	5.122	5.308		5.689	7.231
	deaths	126		105		165	200	207	220		257	380
	expected	(113)		(140)	**	(152)	(177)	(207)	(239)		(280)	(353)
50-54	rate	6.387		9.295		8.001	9.397	9.590	9.310		10.398	12.316
	deaths	138		231		211	296	349	373		424	551
	expected	(166)	*	(226)		(239)	(277)	(347)	(373)		(424)	(522)
55-59	rate	10.495		14.144		14.201	15.942	15.899	16.349		15.665	18.179
	deaths	193		293		337	413	491	587		616	732
	expected	(208)		(300)		(346)	(397)	(499)	(573)		(612)	(726)
60-64	rate	16.733		20.705		24.543	23.257	27.374	27.895		25.277	26.532
	deaths	250		356		482	544	687	844		885	1021
	expected	(229)		(364)		(451)	(571)	(698)	(807)		(923)	(1026)
65-69	rate	22.057		27.839		32.017	35.847	42.112	43.276		42.736	41.764
	deaths	250		374		506	671	932	1044		1240	1414
	expected	(226)		(366)		(504)	(684)	(931)	(1052)		(1215)	(1453)
70-74	rate	23.327		35.895		41.302	51.052	63.705	65.637		69.278	64.449
	deaths	203		343		483	727	1083	1352		1561	1768
	expected	(208)		(335)		(475)	(715)	(1061)	(1346)		(1527)	(1853)
75-79	rate	25.434		41.767		46.659	58.493	84.390	94.314		99.618	109.032
	deaths	147		269		343	556	1000	1392		1811	2205
	expected	(147)		(264)		(375)	(593)	(991)	(1396)		(1801)	(2156)

* P<0.05, **P<0.01. Both were calculated from (obs-exp)^2^/exp.

The diagonals of these tables (from upper left to lower right) define birth cohorts with five-year intervals, each of which is referred to by its central year. For example, individuals aged 30-34 who died in the 1960 calendar year, could have been born at any time between 1926 and 1930; this is the 1928 birth cohort. A total of 17 birth cohorts were obtained: 1883(1881-1885), 1888(1886-1890), …, 1963(1961-1965).

### Statistical Model

Assuming that a hazard model in which the hazard function *λ_k_*(*t*) for the *k*-th cohort (*k*=1, …, *K*) of the proportional form,

$$\lambda_{k}(t)=\exp(\gamma_{k})\exp(\beta_{j})\lambda_{0}(t),$$ (2)

where *t* is the age of the individual, *γ_k_* is the effect due to the *k*-th birth cohort, β*_j_*, is the effect due to the *j*-th period (*j*=1, …, *J*) and *λ*_0_(*t*) is some underlying hazard function. When the number of person-years *N_ij_* in the (*i*, *j*) cell (*i*=1, …, *I*) are fixed, then the numbers of deaths *d_ij_* have independent Poisson distributions and the APC model with additive effects on the logarithmic mortality rate is derived. The parametric form will be:

$$\log\rho_{ij}=\mu +\alpha_{i}+\beta_{j}+\gamma_{k},$$ (3)

$$\sum_{i=1}^{I}\alpha_{i}=\sum_{j=1}^{J}\beta_{j}=\sum_{k=1}^{K}\gamma_{k}=0,$$ (4)

where μ is the overall mean, *α_i_* denotes effect of the *i*-th age group, and *ρ_ij_* = E(*d_ij_*/*N_ij_*). The relation between the three indices *i*,*j* and *k* is given by *k*=*I*-*i*+*j*. This yields *K*=*I*+*J*-1 immediately.

It is well known that the model with all three time factors have suffered from ‘identifiable problem’ due to the relation *k*=*I*-*i*+*j*. To avoid this confusion, the non-linear components (deviation from linearity) of being estimable indices based on Holford^13^^)^ were adopted by removing the linear effects from full effects. For a full effect *α_i_*, the non-linear component (deviation from linearity) of *α_i_* is defined by,

$${\tilde{\alpha }}_{i}=\alpha_{i}-\omega (i,I)\alpha_{0}^{\text{L}},$$ (5)

where *ω*(*x*, *y*) = *x* - (*y*+1)/2 and $\alpha_{0}^{\text{L}}=\sum_{i}\omega (i,I)\alpha_{i}/\sum_{i}\omega (i,I{)}^{2}$ . Using *A_s_*(*i*), (s=1,…,*I*-2) which are orthogonal to the *ω*(*i*, *I*), (i.e. ∑*_i_A_s_*(*i*)*ω*(*i*, *I*) = 0 for every *s*), non-linear components of *α_i_* are also derived to,

$${\tilde{\alpha }}_{i}=\sum_{s=1}^{I-2}A_{s}(i)\alpha_{s}^{N},$$ (6)

where $\alpha_{s}^{N}$ represents the parameter associated with column of the *I*× (*I*-2) design matrix in which each element is A*_s_*(*i*). Nonlinear period and cohort effects ${\tilde{\beta }}_{j}$ , and ${\tilde{\gamma }}_{k}$ are derived in a similar manner with orthogonal components *B_s_*(*i*), and *C_s_*(*i*), respectively. All 2*I*+2*J*-3 parameters in the log linear model were (μ, $\alpha_{0}^{L}$ , $\alpha_{1}^{N}$ , ···, $\alpha_{I\text{-}2}^{N}$ , $\beta_{0}^{L}$ , $\beta_{1}^{N}$ , ···, $\beta_{J\text{-}2}^{N}$ , $\gamma_{0}^{L}$ , $\gamma_{1}^{N}$ , ···, $\gamma_{K\text{-}2}^{N}$ ), with corresponding overall design matrix being (1, *ω*(*i*, *I*), *A*_1_(*i*), ···, *A_I_*_-2_(*i*), *ω*(*j*, *J*), *B_I_*(*i*), ···, *B_J_*_-2_(*i*), *ω*(*k*, *K*), *C_t_*(*i*), ···, *C_K_*_-2_(*i*)) for any *i*th row. Here we obtained the APC model with non-linear components,

$$\begin{array}{rl} \log\rho_{ij} & =\mu +({\tilde{\alpha }}_{i}+\omega (i,I)\alpha_{0}^{L})+({\tilde{\beta }}_{j}+\omega (j,J)\beta_{0}^{L})+\\ & \;({\tilde{\gamma }}_{k}+\omega (k,K)\gamma_{0}^{L}), \end{array}$$ (7)

The identifiable problem extracted here is that if $\alpha_{0}^{L}$ , $\beta_{0}^{L}$ and $\gamma_{0}^{L}$ were a solution of linear trends of the APC model then $\alpha_{0}^{L}+t$ , $\beta_{0}^{L}\text{-}t$ and $\gamma_{0}^{L}+t$ could be another solution for arbitrary *t*. There arises the problem of identifiable parameters, whereas all non-linear components and the following combination of linear trends ${\text{d}}_{1}\alpha_{0}^{L}+{\text{d}}_{2}\beta_{0}^{L}+({\text{d}}_{2}{\text{-d}}_{1})\gamma_{0}^{L}$ for arbitrary d_1_, d_2_ were shown to be estimable. The maximum likelihood method was used to estimate parameters in this study. The goodness of fit was examined by the likelihood ratio statistic *G*^2^ and by predicted values of death numbers.

## RESULTS

The APC model gave a predicted value (in parentheses) in each cell as shown in Table 1 in males and Table 2 in females. The differences between observed and expected numbers of deaths from lung cancer were not excessive. Five out of eighty cells gave significant differences (*P*<0.05) in both genders. Only one cell was *P*<0.01 in males and two were in females. The maximum of the differences between observed and expected numbers were 107(2.2% of expected value) in the cell of age 70-74 in 1990 in males, 85(4.6%) in the cell of age 70-74 in 1995 in females. The deviance was 81.6 with 48 degrees of freedom in males (*P*=0.001), and 97.6 with 48 degrees of freedom in females (*P*<0.0001). The *χ*^2^ values were significant, however this was partly due to the large number of deaths which enable us to detect even small departures from the model. Table 3 indicates a summary of the data when a subset of the factors is used. The proportion of the lack of fit which was not explained by age was 99.1% in males and 94.9% in females for the age-period-cohort models, as shown by $R_{A}^{2}$ in Table 3.

**Table 3.** Summary chi square of age(A), period (P) and birth cohort(C) models for data in Table 1 and Table 2.

Model	Males				Females			

	df	G^2^	R_A_^2^	G^2^/df	df	G^2^	R_A_^2^	G^2^/df
A,P,C	48	81.589	0.991	1.700	48	97.562	0.949	2.033
A,P	63	1223.824	0.993	19.426	63	519.567	0.731	8.247
A,C	54	140.462	0.985	2.601	54	118.451	0.939	2.194
A	70	9251.807			70	1929.221		

Estimated parameters derived from the APC model were shown in Table 4. Age 30-34, calendar year 1960 and birth cohort 1883 was set as a standard criterion for the comparison. Linear trends of $\alpha_{0}^{L}$ , $\beta_{0}^{L}$ and $\gamma_{0}^{L}$ could not be separated, however projection by extinguishing one of three effects eased interpretation. Linear effects of age and period and their nonlinear effects were obtained under the assumption that linear birth cohort effects were zero. Non-linear effect expresses the original change pattern in each effect, and full effect which is the sum of linear and non-linear effect expresses trends of the risk of lung cancer. The patterns of non-linear age effects were convex with maximal effects laying on the 55-59 and 60-64 age categories in males and aged 50-54 in females. Non-linear age effects were considered small enough to neglect compared with their linear effects. Non-linear period effects were originally small and close to zero. Full effects of age and period were considered to change linearly. To compare the gradients, the increasing risk of age is 6.45 times greater in males and 6.56 times greater in females than that of period.

**Table 4.** Age, period and cohort effects for age-period-cohort model.


Males														

						Calender year							

						1960	1965	1970	1975	1980	1985	1990	1995	

Period effects(L)						0.000	0.128	0.256	0.384	0.511	0.639	0.767	0.895	

Period effects(N)						0.000	0.003	-0.013	-0.006	0.034	0.035	0.016	-0.036	

Period effects						0.000	0.130	0.243	0.377	0.546	0.674	0.783	0.859	

Age (years)	AGE(L)		AGE(N)	Age effects		Birth cohort effects(N)							

30-34	0.000		0.000	0.000		0.779	0.643	0.466	0.534	0.589	0.529	0.345	0.433	
35-39	0.622		0.273	0.895		0.778	0.779	0.643	0.466	0.534	0.589	0.529	0.345	
40-44	1.244		0.412	1.656		0.838	0.778	0.779	0.643	0.466	0.534	0.589	0.529	
45-49	1.866		0.498	2.364		0.854	0.838	0.778	0.779	0.643	0.466	0.534	0.589	
50-54	2.488		0.553	3.040		0.881	0.854	0.838	0.778	0.779	0.643	0.466	0.534	
55-59	3.110		0.573	3.683		0.873	0.881	0.854	0.838	0.778	0.779	0.643	0.466	
60-64	3.732		0.563	4.295		0.702	0.873	0.881	0.854	0.838	0.778	0.779	0.643	
65-69	4.354		0.467	4.821		0.515	0.702	0.873	0.881	0.854	0.838	0.778	0.779	
70-74	4.975		0.272	5.247		0.331	0.515	0.702	0.873	0.881	0.854	0.838	0.778	
75-79	5.597		-0.053	5.544		0.000	0.331	0.515	0.702	0.873	0.881	0.854	0.838	

L denotes linear effects and N denotes non-linear effects. μ =-12.653.														
Means of non-linear age, period and cohort effects are 0.356, 0.004 and 0.593, respectively.														

Females														

					Calender year									

					1960		1965	1970	1975	1980	1985	1990	1995	

Period effects(L)					0.000		0.091	0.181	0.272	0.362	0.453	0.543	0.634	

Period effects(N)					0.000		0.095	0.026	0.012	0.078	0.050	0.023	0.032	

Period effects					0.000		0.185	0.208	0.284	0.441	0.503	0.566	0.666	

Age (years)	AGE(L)	AGE(N)		Age effects	Birth cohort effects(N)									

30-34	0.000	0.000		0.000	0.588		0.501	0.547	0.563	0.543	0.462	0.256	0.383	
35-39	0.479	0.271		0.750	0.664		0.588	0.501	0.547	0.563	0.543	0.462	0.256	
40-44	0.957	0.290		1.247	0.767		0.664	0.588	0.501	0.547	0.563	0.543	0.462	
45-49	1.436	0.397		1.832	0.794		0.767	0.664	0.588	0.501	0.547	0.563	0.543	
50-54	1.914	0.451		2.365	0.810		0.794	0.767	0.664	0.588	0.501	0.547	0.563	
55-59	2.393	0.424		2.816	0.750		0.810	0.794	0.767	0.664	0.588	0.501	0.547	
60-64	2.871	0.384		3.255	0.614		0.750	0.810	0.794	0.767	0.664	0.588	0.501	
65-69	3.350	0.293		3.643	0.488		0.614	0.750	0.810	0.794	0.767	0.664	0.588	
70-74	3.828	0.192		4.021	0.292		0.488	0.614	0.750	0.810	0.794	0.767	0.664	
75-79	4.307	0.067		4.374	0.000		0.292	0.488	0.614	0.750	0.810	0.794	0.767	

L denotes linear effects and N denotes non-linear effects. μ =-12.651.														
Means of non-linear age, period and cohort effects are 0.277, 0.040 and 0.531, respectively.														

**Figure 1.**
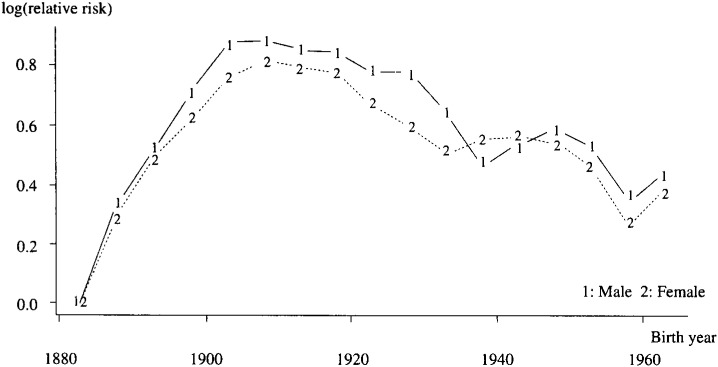
Non-linear birth cohort effects from APC model by gender.

Non-linear birth cohort effects indicated interesting patterns (Figure 1). These effects increased until the 1908 birth cohort in both genders. The parameters decreased from the 1908 birth cohort to 1938 in males and 1933 in females. They had a second increase period from 1938 to 1948 in males, and 1933-1943 in females. They decreased and changed upward at 1958 again. The birth cohort effect was observed to have five points of change in both genders.

**Figure 2.**
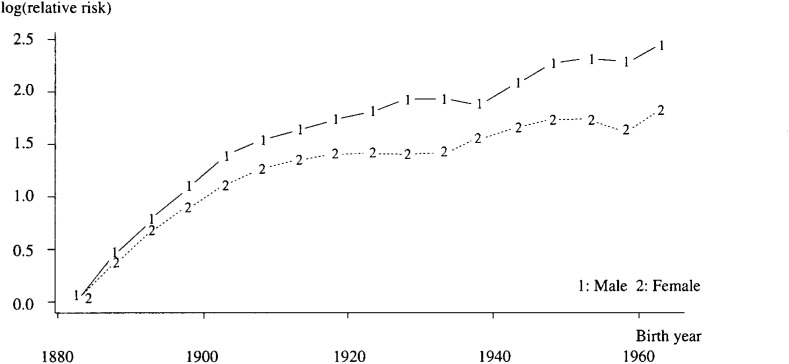
Full birth cohort effects from APC model by gender.

If linear period effects were assumed to be zero, the corresponding cohort effect was obtained. These effects were also peculiar in pattern even in the full effects (Figure 2)*.* The pattern also changed five times: 1903,1928,1938,1948 and in males and 1908,1918,1933,1943 and 1958 in females.

In both genders, the trends of non-linear and full birth cohort effect means: (1) The latent risk in the birth cohort has increased since 1883, (2) The birth cohort effect has five points of change, (3) The change in the 1963 birth cohort effect seemed to increase differently from previous birth years.

## DISCUSSION

It is unfortunate that Japan has not started a cancer registry system at the national level. As a result, for a longer trend analysis, mortality seems to be the only indicator. The most prominent weakness of mortality data is the impossibility of separating factors of incidence and improvement of medical technology (length of case-fatality). In the case of lung cancer, incidence rate and mortality rate are considered to be almost parallel because of the little improvement in medicine for survival.

In the APC model, the interpretation of changes of pattern of birth cohort effects is difficult. One way to interpret is to observe the pattern of change of non-linear effects.

Full effects of age and period were shown to change linearly because of small influence of their non-linear effects compared with linear ones. Averaged change rate of full effects of age and period by moving five years were 0.544 and 0.107, respectively. Increasing risk by aging was almost five times larger than that by changing period. In contrast, non-linear birth cohort effects had enough values not to be neglected. The birth cohort effects had unique patterns, differently from age and period effects.

Non-linear birth cohorts between the periods with minimal values and maximal values (1938-1948 in males and 1933-1943 in females) had peculiar patterns. Persons who were born in those periods had their main growth period in the middle of World War II (1940-1945). There may be some characteristics for these changing patterns. The first pattern, before 1903 in males and before 1908 in females, might be considered rapid modernization in Japan in the Meiji era. The second pattern of change to decrease in 1903-1938 in males and in 1908-1933 in females, were considered as the first success of activities of promoting hygiene and public health in Japan. The third pattern of change to increase in 1938-1948 in males and in 1933-1943 in females, might be due to wars in the early Showa era. The peculiarity in this period was also reported for diabetes mellitus, ischemic heart disease, cirrhosis of the liver and suicide^34^^)^. The fourth pattern of decrease in 1948-1958 in males and in 1943-1958 in females might be improvement of hygiene and public health after World War II. The fifth pattern after 1958 should be interpreted cautiously. After 1960, Japan faced a so-called ‘high speed economic growth period’. Because of the high industrial growth in this period, the environmental pollution by chrome, nickel, asbestos and nitrosamines were higher level than at present, (but the nitrosoxide level has remained almost a constant level)^35^^)^. Many everyday foods containing chemical substances with high fat and high cholesterol^36^^)^ have also emerged in this period. Based on these points, monitoring may be necessary for 1958 birth cohort and later. In this analysis, however, the effect of 1958 birth cohort is the last time frame that was analysed. The possibility that the result was caused by a random fluctuation cannot be denied, because the effect of the 1958 birth cohort was only estimated from the one cell, that was 30-34 years old in 1995.

To analyse temporal trends of mortality and incidence rates, APC analysis is one of the more popular tools. However conventional APC analysis has suffered from the identifiable problem, which causes difficulties with interpretation of the results, because of an unreasonable additional assumption. The method to separate common linear effects and original non-linear effects is reasonable in the technical sense. To interpret the results is still not easy, however, meaningful reasonable results can be obtained. Conventional univariate analysis based on standardised mortality ratio (SMR) or comparative mortality figure (CMF) in period effects (differences of both indices were examined^37^^)^ and standardised cohort mortality ratio (SCMR) or comparative cohort mortality figure (CCMF) in birth cohort effects is the other tools. These methods clarify the trend of only one of the effects by using the above annual indices. So these results have been believed to easiliy interpreted. However these methods could not separate the three linear effects of age period and cohort.

Hamajima et al^38^^)^ developed another model based on a Weibull hazard function with each birth cohort effect as a constant to analyse the single birth cohort effect and to predict the future risks. According to the Hamajima model, the birth cohort effect of 1896-1900 and 1931-35 were 0.0805 and 0.2181, respectively. The corresponding logarithms of the full birth cohort effects in males in the APC model were 1.0854 and 1.9209, respectively. The increasing birth cohort effect is 2.57 times in the Hamajima model, and 2.31 times in our model, which are almost equivalent. The advantages in using the APC model compared to his model are: (1)The assumption in our model is less strict meaning not to have common mortality distribution on each year,(2) The birth cohort effects in our model are estimated by considering the other two effects simultaneously.

**Figure 3.**
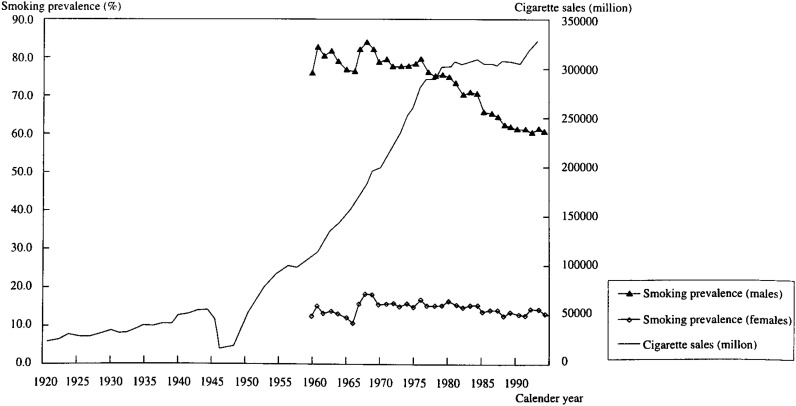
The trend of national cigarette sales and smoking prevalence in Japan.

As is well known, lung cancer is related to cigarette smoking. Recently the quantitative relationship between cumulative cigarette consumption and lung cancer mortality was recognized by linear regression analysis between the estimated adjusted cumulative cigarette consumption and the lung cancer death rate in each age group (20-24,…,70-74)^39^^)^. Consideration between cigarette smoking and lung cancer based on our model is interesting. With the exception of the period during World War II, cigarette sales in Japan have steadily increased and smoking prevalence has been decreasing in males and almost constant in females since the 1960’s when statistics started to be gathered(Figure 3). To simplify this situation, annual smoking amounts can be estimated by the product of national cigarette sales and smoking prevalence. The respective vales were 107.4 billion, 75.9% in males, 12.4% in females in 1960, and 328.9 billion, 60.4% in males and 13.3% in females in 1992. These logarithms of changes are 0.89 in males and 1.19 in females. Our period effect in 1995 is 0.859 in males and 0.666 in females. In males the APC model was considered to support the relation. The factors that cause this difference in females might be the daily smoking amounts per day or the method of inhaling.

Another interesting problem is that of area difference. In our model we consider Japan as a unit, though there is considered to be area variation. According to Kano et al^40^^)^, Saitama males and Fukushima females had the highest lung cancer death rate in 1960 and Okinawa males and Oita females in 1980. The major change of age-adjusted death rates from 1960-1980 were observed in Kagoshima, Tokushima and Wakayama, in males; and Shimane, Oita and Kagawa in females.

APC analysis based on estimable function could separate linear effects and non-linear effects. Linear effects are recognized as common effects (impossible to separate). Non-linear effects are original effects of time factors. The best way to interpret the APC model at the present is to reveal changing patterns with estimable parameters, and if necessary, to add linear effects obtained by one of three effects extinguished to zero. This method shows that the APC analysis produces more precise and reasonable results than conventional analyses (summarised indices or two parameters AP, AC model). Estimators from conventional analyses have non-negligible problems: (1) The pattern depends on the ways of taking a standard population, (2) Incomplete cells based on missing values in the birth cohort gave less precise SCMRs (CCMFs), (3) Two way model (age-period, or age-cohort) gave no information for the other component. The APC model overcomes these ambiguities. So, this model is considered effective although there have been many criticisms.

## CONCLUSION

The mortality of lung cancer in Japan based on the APC model revealed that there are five changing patterns in the birth cohort effects in Japanese lung cancer mortality. The 1938, 1943 and 1948 birth cohort in males and 1933, 1938 and 1943 birth cohort in females were peculiar patterns. Successive birth cohorts need to be monitored for increased risk. Future prevention activities will be based on need, if risk increases in these birth cohorts.
